# Correction to: Regeneration Rosetta: An Interactive Web Application To Explore Regeneration-Associated Gene Expression and Chromatin Accessibility

**DOI:** 10.1093/g3journal/jkaf026

**Published:** 2025-04-16

This is a correction to: Andrea Rau, Sumona P Dhara, Ava J Udvadia, Paul L Auer, Regeneration Rosetta: An Interactive Web Application To Explore Regeneration-Associated Gene Expression and Chromatin Accessibility, *G3 Genes|Genomes|Genetics*, Volume 9, Issue 12, 1 December 2019, Pages 3953–3959, https://doi.org/10.1534/g3.119.400729

Supporting data originally available at https://ls-shiny-prod.uwm.edu/rosetta/ is now available at https://rosetta.sk8.inrae.fr/.

